# Comparison of model predictions of typhoid conjugate vaccine public health impact and cost-effectiveness

**DOI:** 10.1016/j.vaccine.2022.12.032

**Published:** 2023-01-23

**Authors:** Holly Burrows, Marina Antillón, Jillian S. Gauld, Jong-Hoon Kim, Vittal Mogasale, Theresa Ryckman, Jason R. Andrews, Nathan C. Lo, Virginia E. Pitzer

**Affiliations:** a Yale School of Public Health, Yale University, New Haven, CT, USA; b Swiss Tropical and Public Health Institute, Allschwil, Switzerland; c University of Basel, Basel, Switzerland; d Institute for Disease Modeling, Bill & Melinda Gates Foundation, Seattle, WA, USA; e Public Health, Access, and Vaccine Epidemiology (PAVE) Unit, International Vaccine Institute, Seoul, Republic of Korea; f Policy and Economic Research Department, International Vaccine Institute, Seoul 08826, Republic of Korea; g Department of Epidemiology, Johns Hopkins Bloomberg School of Public Health, Baltimore, MD, USA; h Division of Infectious Diseases and Geographic Medicine, Stanford University School of Medicine, Stanford, CA, USA; i Division of HIV, Infectious Diseases, and Global Medicine, University of California, San Francisco, San Francisco, CA, USA

**Keywords:** Typhoid fever, Mathematical modeling, Model comparison, Typhoid conjugate vaccines, Economic evaluation

## Abstract

Models are useful to inform policy decisions on typhoid conjugate vaccine (TCV) deployment in endemic settings. However, methodological choices can influence model-predicted outcomes. To provide robust estimates for the potential public health impact of TCVs that account for structural model differences, we compared four dynamic and one static mathematical model of typhoid transmission and vaccine impact. All models were fitted to a common dataset of age-specific typhoid fever cases in Kolkata, India. We evaluated three TCV strategies: no vaccination, routine vaccination at 9 months of age, and routine vaccination at 9 months with a one-time catch-up campaign (ages 9 months to 15 years). The primary outcome was the predicted percent reduction in symptomatic typhoid cases over 10 years after vaccine introduction. For three models with economic analyses (Models A-C), we also compared the incremental cost-effectiveness ratios (ICERs), calculated as the incremental cost (US$) per disability-adjusted life-year (DALY) averted. Routine vaccination was predicted to reduce symptomatic cases by 10–46 % over a 10-year time horizon under an optimistic scenario (95 % initial vaccine efficacy and 19-year mean duration of protection), and by 2–16 % under a pessimistic scenario (82 % initial efficacy and 6-year mean protection). Adding a catch-up campaign predicted a reduction in incidence of 36–90 % and 6–35 % in the optimistic and pessimistic scenarios, respectively. Vaccine impact was predicted to decrease as the relative contribution of chronic carriers to transmission increased. Models A-C all predicted routine vaccination with or without a catch-up campaign to be cost-effective compared to no vaccination, with ICERs varying from $95–789 per DALY averted; two models predicted the ICER of routine vaccination alone to be greater than with the addition of catch-up campaign. Despite differences in model-predicted vaccine impact and cost-effectiveness, routine vaccination plus a catch-up campaign is likely to be impactful and cost-effective in high incidence settings such as Kolkata.

## Highlights

• While model predictions varied, typhoid conjugate vaccines are likely cost-effective.
• Differing structural assumptions can explain discrepancies in model predictions.
• Static model estimates of vaccine impact are not necessarily conservative.
• Increasing the contribution of chronic carriers decreased predicted vaccine impact.

## 1 Introduction

Typhoid fever is caused by the human-restricted bacterial pathogen *Salmonella enterica* serovar Typhi (*S*. Typhi) and is transmitted primarily through fecal contamination of food and water. Although improvements in water, sanitation, and hygiene (WASH) have promoted declines in the global burden of typhoid, there were still an estimated 10.9 million cases in 2017 [1], the vast majority of which occurred in low- and lower-middle-income countries that often lack adequate WASH infrastructure [2], [3]. While treatment with antibiotics is generally effective, prevalence of antimicrobial resistance is increasing in many endemic countries [4]. Vaccination may be a cost-effective approach to typhoid control in regions where improvements in WASH are unsustainable or unlikely to occur in the near-term.

In recent years, typhoid conjugate vaccines (TCVs) have been developed to address the shortcomings of existing live-attenuated and polysaccharide vaccines. Recent randomized trials have found TCVs to be highly effective in reducing risk of typhoid fever [5], [6], [7]. The World Health Organization now recommends TCVs over other typhoid vaccines at all ages due to their improved immunological properties, suitability for use in young children, and longer expected duration of protection [8], [9]. Three TCVs have been licensed in India, two of which are WHO prequalified, with additional vaccines under development [10]. Many countries are now considering whether and how to incorporate TCVs into national immunization programs, and questions of TCV impact and cost-effectiveness are critical to decision-making and vaccine prioritization.

Models to evaluate vaccine impact and cost-effectiveness often differ in their construction due to divergences in epidemiological assumptions, model parameterization, and approaches to capturing processes such as the natural history of disease or human behavior; these processes are often not fully understood and/or need to be simplified [11], [12], [13]. Furthermore, models may make different assumptions about the economic costs of vaccine delivery, treatment, and health outcomes. Discrepant model predictions, such as those from previous economic evaluations of TCVs, can be difficult to interpret, particularly when formulating policy recommendations [12], [14], [15]. Although uncertainty in model parameters is commonly explored within a single model using sensitivity analyses, differences in model structure are not often addressed [13]. Model comparison exercises can explore how differences in model structure among groups of disease models affect predictions for the potential impact of interventions [16], [17], [18], [19].

In this analysis, we compared five models of TCV impact identified through a literature search and consultation with experts. The models were fitted to a common dataset from Kolkata, India, which is an endemic high-incidence setting [20]. We compared model approaches and resulting predictions to provide robust estimates for TCV impact that account for model differences to determine the degree to which structural uncertainty impacts cost-effectiveness of TCV delivery strategies.

## 2 Methods

### 2.1 Description of the models

Of the five models identified that predicted the impact of TCV delivery strategies on typhoid fever, four were dynamic and one was static (Table 1). Two of the dynamic models (Models A-B) and the static model (Model C) also evaluated the cost-effectiveness of TCV introduction.

**Table 1:** Description of key features of five models.

**Model Feature**	**Model A**	**Model B**	**Model C**	**Model D**	**Model E**
**Model type**	Dynamic, age-structured, deterministic	Dynamic, age-structured, deterministic	Static, decision-tree model	Dynamic, age-structured, deterministic	Dynamic, individual-based, stochastic
**Infection states**	S + I + R + C + V + W	S_1_ + I_1_ + R + S_2_ + I_2_ + C + V_1_ + V_2_ + W	NA	S + I + R + C + V + W	U + S + Inc + AI/SI + C + W + V
**Environmental state**	Yes	Yes	No	Yes	Yes
**Duration of chronic carriage**	10 years	Life-long	NA	Life-long	Life-long
**Natural immunity**	Waning	Waning	NA	Waning	Leaky/waning immunity, based on number of previous infections
**Estimated parameters**	Short-cycle transmission coefficient Long-cycle transmission coefficient Duration of immunity Proportion of infections that are symptomatic Relative risk of infection of < 5 yr olds	Basic reproductive numberRate of transition from “susceptible to subclinical reinfection” (S2) to “fully susceptible” (S1) Proportion of infections that are symptomatic Relative risk of infection of < 2 yr olds Relative risk of infection for 2–4 yr olds	NA	Basic reproductive number Duration of immunity Proportion of infections that are diagnosed Relative risk of infection for < 2 yr olds Relative risk of infection for 2–4 yr olds	Exposure rate through the short cycle Exposure rate through the long cycle Reduction in susceptibility after infection Duration of immunity Probability of infection being symptomatic Age-specific exposure curve

Three of the dynamic models (Models A, B, and D) were age-structured, deterministic, differential-equation-based compartmental models that followed a SIRS-like (susceptible-infectious-recovered-susceptible) framework, and one (Model E) was a stochastic individual-based model. The SIRS-like models divided the population into four infection states: *S,* susceptible to infection; *I,* infected and infectious; *R,* recovered and temporarily immune; and *C*, chronic carriers. An age-dependent fraction of infected individuals (*I*) is assumed to develop chronic carriage and enter the *C* compartment, a fraction is assumed to die and leave the model, while the remaining individuals develop immunity to reinfection and enter the *R* compartment. Immunity is assumed to wane over time, and recovered individuals (re)enter a susceptible compartment (*S*). Compartments for vaccinated individuals (*V*) and water-borne bacteria (*W*) were also included.

The dynamic models all assume immunity from vaccination is “all-or-nothing” (i.e., sterilizing) and wanes over time. A fraction of vaccinated individuals equal to the vaccine coverage times initial vaccine efficacy (*VE*_0_) is assumed to enter the vaccinated state (*V*) where they are fully protected from infection; the remaining (1-*VE*_0_) vaccinated individuals remain in the susceptible state. Vaccine-induced immunity is assumed to wane, such that vaccinated and protected individuals return to the susceptible state (*S*) at a rate *ω_v_* inversely proportional to the average duration of protection.

The compartmental diagrams are shown in Figure S1. Estimated parameters varied by model (Table 1). Certain parameters describing the natural history of typhoid infection were fixed to a common set of values derived from the literature (Table 2).

**Table 2:** Common parameter assumptions for the dynamic models.

**Parameter definition**	**Value**	**Source/Notes**
***Demographic parameters***		
Birth rate (per year)	25.3 live births per 1,000	[21]
Death rate (per year)	0.001–0.0479 (varies by age; see Table S1)	[21]
Population size	100,000 (see Table S1 for age distribution)	Assumption
***Disease parameters***		
Mean duration of infectiousness	4 weeks	[22]
Fraction infected who become carriers	0.003–0.092 (varies by age; see Table S1)	[23]
Disease-induced mortality rate (fraction of symptomatic cases that die)	0.01	[1], [24]
Proportion of symptomatic cases that seek care	0.75	[25], [26]
Sensitivity of blood culture for diagnosing typhoid fever (reporting fraction for *clinical* cases)	0.6	[27], [28]
Rate of shedding into the water supply	1 infectious unit/week	Assumption (inseparable from *β_w_* in Models A, B, D); fitted for Model E
Rate of decay of infectious particles from water supply	1/3 week^−1^	[29], [30]
Proportion of transmission occurring via long-cycle	0.5	Assumption for Models B, D; fitted for Models A, E
Relative infectiousness of acute subclinical infections	0.5	Applies to Models B and E only [31]
Relative infectiousness of chronic carriers	0.01, 0.1, 0.5	Examined 3 different assumptions in scenario analyses

Model A, based on Lo et al. [15], is an SIRS-like model that includes “short-cycle” transmission (e.g., person-to-person) proportional to the prevalence of infectious individuals in *I* and *C*, and “long-cycle” transmission (e.g. water-borne) from the shedding of bacteria into an environmental reservoir (*W*). Unlike the other dynamic models, Model A assumes chronic carriers recover into the *R* state after a mean duration of 10 years, based on the assumption that carriage may not be lifelong (e.g., due to antibiotic use).

Model B is based on Pitzer et al. [32]. It divides the SIR compartments into six separate infection states: *S*_0_, fully susceptible to typhoid infection; *I*_1_, infected with the potential for symptomatic illness and diagnosis with clinical disease; *R*, recovered from infection and temporarily immune to reinfection; *S*_1_, susceptible to subclinical typhoid infection; *I*_2_, subclinically infected with typhoid. Chronic carriers (*C*) remain infectious for life. Susceptible individuals can become infected through short-cycle (from *I*_1_, *I*_2_, and *C*) or long-cycle transmission from the environmental state (*W*). The model also differentiates between fully susceptible individuals who are vaccinated and protected (*V*_1_), and those who were vaccinated after a previous infection (*V*_2_); individuals return to the respective susceptible state (*S*_1_ or *S*_2_) following the waning of vaccine-induced immunity.

Model C is a static cohort-based decision-tree model that uses assumptions regarding the population demographics, typhoid fever incidence, care-seeking and treatment possibilities, and case fatality risk (CFR) to estimate the impact of vaccination. The model was developed by the International Vaccine Institute (IVI) as part of the TCV investment case in 2014. The cohort model divides the population into 1-year age groups (from 0 to 50 + years of age). The number of individuals in each age group was informed by the person-time of follow-up in the Sur et al. study [33], and all individuals were assumed to age into the next age group after one year (i.e. there was no mortality except from the last age group). The number of symptomatic typhoid fever cases in each age group was equal to the observed incidence rate of typhoid fever times the population size. Vaccine impact was modeled as the vaccination coverage times vaccine efficacy for each year of follow-up, where the efficacy *y* years after vaccination was modeled as: $VE_{y}=VE_{0}*exp\left(-\omega_{v}y\right)$, where *VE*_0_ is the initial vaccine efficacy and *ω_v_* is the rate of waning vaccine-induced immunity.

Model D is a dynamic model developed by researchers from IVI. It is similar to Model A, but chronic carriers are assumed to remain infectious until they die, and individuals who are recovered and immune from previous infection are assumed to re-enter the fully susceptible state following waning of immunity. For model fitting, the demographic parameters were held constant and the remaining parameters were estimated by likelihood-based Markov Chain Monte Carlo.

Model E, based on Gauld et al. [34], is different from the other dynamic models in that it is a stochastic, individual-based model. Infections can be acute or subclinical, with the inclusion of a life-long chronic carrier state that can result from both types of infections. The model also includes an “unexposed” state into which individuals are born prior to becoming susceptible as they age, as well as a latent period following infection prior to symptom onset. Transmission can occur via the short- and long-cycle, and a dose–response relationship is used to characterize long-cycle transmission. Natural immunity is assumed to be “leaky”, such that individuals with immunity from previous infection can be reinfected, but at a reduced rate compared to fully susceptible individuals; the relative risk of reinfection depends on the number of previous infections. The model was fitted using a gradient ascent algorithm.

### 2.2 Data sources and common model parameters

We fit the models to a common dataset from Kolkata, India (Table S1), due to the availability of published data on the population-based incidence of typhoid fever and cost of treatment. Typhoid fever incidence data were collected as described by Sur et al. [20]. In brief, outpost-based surveillance was conducted January 1 to December 31, 2004. Typhoid fever was defined as an episode of fever lasting three or more days in which *S.* Typhi was isolated by blood culture. The population residing in the catchment area at the time of a census in early 2004 was used as the denominator for estimating incidence; each person in the catchment area was assumed to contribute 12 months of person-time [20]. The unadjusted incidence of blood-culture-confirmed typhoid fever was 160 cases per 100,000 person-years [20].

Assumptions about vaccine efficacy (VE) and duration of protection were based on models fitted to previously available estimates for the Vi-rEPA conjugate vaccine over 46 months of follow-up during a trial conducted in Vietnam [35], [36]. Since there is uncertainty about the efficacy and duration of protection of different TCV candidates, we explored a variety of assumptions. Under an “optimistic scenario”, the initial efficacy (i.e., vaccine “take”) was assumed to 95 % and the average duration of protection (inverse of the waning rate) was assumed to be 19 years, as estimated from follow-up data for the Vi-rEPA vaccine. For the “pessimistic scenario”, we assumed an initial efficacy of 82 % and an average duration of protection of 6 years, based on the lower 95 % confidence interval of estimates for the Vi-rEPA vaccine [26], [35], [37], [38]. We also explored scenarios assuming an initial efficacy of 95 % and 6-year duration of protection and initial efficacy of 82 % and 19-year duration of protection. We validated our assumptions by comparing to more recent estimates for the Typbar-TCV (Figure S2) [5], [6], [7].

Treatment cost data were based on two Kolkata-based studies by Sur at al. and Poulos et al. [33], [39]. Both studies used a micro-costing bottom-up approach to calculate the unit cost of medical services. Sur et al. conducted a facility-based cost-of-illness analysis of direct medical costs, which were calculated by multiplying the quantity of medical services consumed by 83 typhoid patients by their unit costs [33]. Poulos et al. calculated community-based estimates of both the health facility and patient-borne costs of blood-culture-confirmed typhoid fever [39]. Direct and indirect (i.e., lost wages and productivity) patient-borne costs were surveyed from 79 culture-confirmed typhoid patients [39].

We assumed 75 % of cases would seek treatment for their illness; 8 % of these individuals would be hospitalized and the remaining 92 % would be treated as outpatients [25]. Among hospitalized inpatients, we assumed 82 % would have no complications, while 18 % would experience intestinal perforation or other complications. The case fatality risk (CFR) was assumed to be 11 % among inpatients who experienced complications, while all other cases were assumed to have a CFR of 1 %. We also explored scenarios in which the CFR among uncomplicated cases was 0.5 %.

### 2.3 Vaccination strategies and impact measures

Vaccination was incorporated into each of the calibrated models, and the impact of vaccination under the four assumptions regarding vaccine efficacy were explored. We evaluated the impact of two TCV delivery strategies compared to a no-vaccination scenario: routine vaccination with one dose of TCV at 9 months of age, and routine vaccination plus a one-time catch-up campaign among all individuals 9 months to < 15 years of age. We assumed a fixed coverage of 85 % for routine vaccination and 75 % for the catch-up campaign.

The impact of vaccination was measured as the percent reduction in the cumulative incidence of symptomatic typhoid fever cases over a 10-year period following TCV introduction. We also compared the projected annual incidence of typhoid fever cases per 100,000 population for each vaccination strategy and under each combination of vaccine efficacy assumptions across the 10-year timespan. The 10-year time horizon was chosen to reflect the approximate duration of vaccine-induced immunity across the different scenarios, to allow for comparison with previously published typhoid vaccine cost-effectiveness analyses [14], [15], and to reflect the inherent uncertainty in the dynamics of infectious diseases decades into the future.

### 2.4 Cost-effectiveness analysis

We calculated and compared incremental cost-effectiveness ratios (ICERs), measured as the incremental cost (in US$) per disability-adjusted life-year (DALY) averted, based on the predicted vaccine impact output from Models A-C. Common assumptions regarding treatment probabilities, health outcomes, and costs are outlined in Supplementary Table 2. A discount rate of 3 % per year was applied to both the costs and effects. Strategies were considered strongly dominated if they cost more and provided fewer benefits (i.e., DALYs averted) than another strategy, and strategies were considered weakly dominated if the ICER for a strategy was higher than the next most expensive intervention. Dominated strategies represent inefficient choices.

### 2.5 Sensitivity analyses

It has been previously shown that the predicted level of indirect and overall protection depends on the proportion of transmission due to chronic carriers [32]. Therefore, we assessed vaccine impact under three different assumptions for the relative infectiousness of chronic carriers, *r =* 0.01, 0.1 (base case), and 0.5.

For Models B and E, we also performed a sensitivity analysis of the predicted vaccine impact (percent reduction in symptomatic typhoid fever cases) and projected annual incidence of typhoid fever cases (per 100,000 population) assuming vaccine-induced immunity was “leaky” rather than “all-or-nothing”. For the “leaky” scenario, we assumed all vaccinated individuals received some protection upon vaccination, but the risk of infection for individuals in the vaccinated state (*V*) was reduced by a proportion equal to the initial vaccine efficacy (*VE*_0_).

Finally, for Models A-C, we also estimated the cost-effectiveness of TCV introduction assuming the overall incidence of typhoid fever was 80 % lower than in our main analysis (i.e. an unadjusted incidence of 32 cases per 100,000 person-years). We assumed the incidence was reduced by the same percentage across all age groups.

## 3 Results

**Fig. 1:**
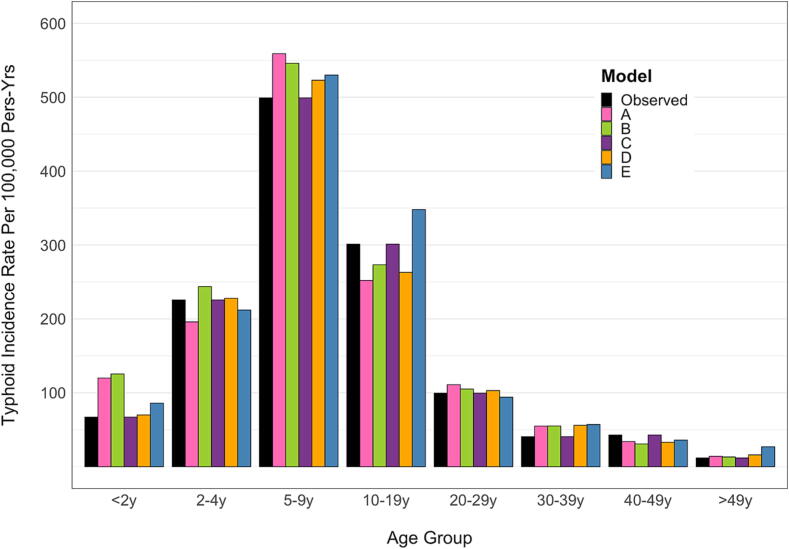
Model fits to the incidence of blood-culture-confirmed typhoid fever during a population-based surveillance study in Kolkata, India. The black bars represent the observed incidence [20], while the colored bars represent the fitted models. Note that Model C is a static model that uses the observed age-specific incidence as an input.

All fitted models were qualitatively similar to the age-stratified Kolkata data (Fig. 1). Models A and B overestimated typhoid incidence in the < 2-year age group, while Model E overestimated incidence in the ≥ 50-year age group. Model D most closely resembled the data, while Model C used the age-specific incidence as a model input and thus directly reproduced the observed age-specific incidence data prior to vaccine introduction. Estimated parameters for each model are described in Table S3.

### 3.1 Vaccine impact over a 10-year timespan

**Fig. 2:**
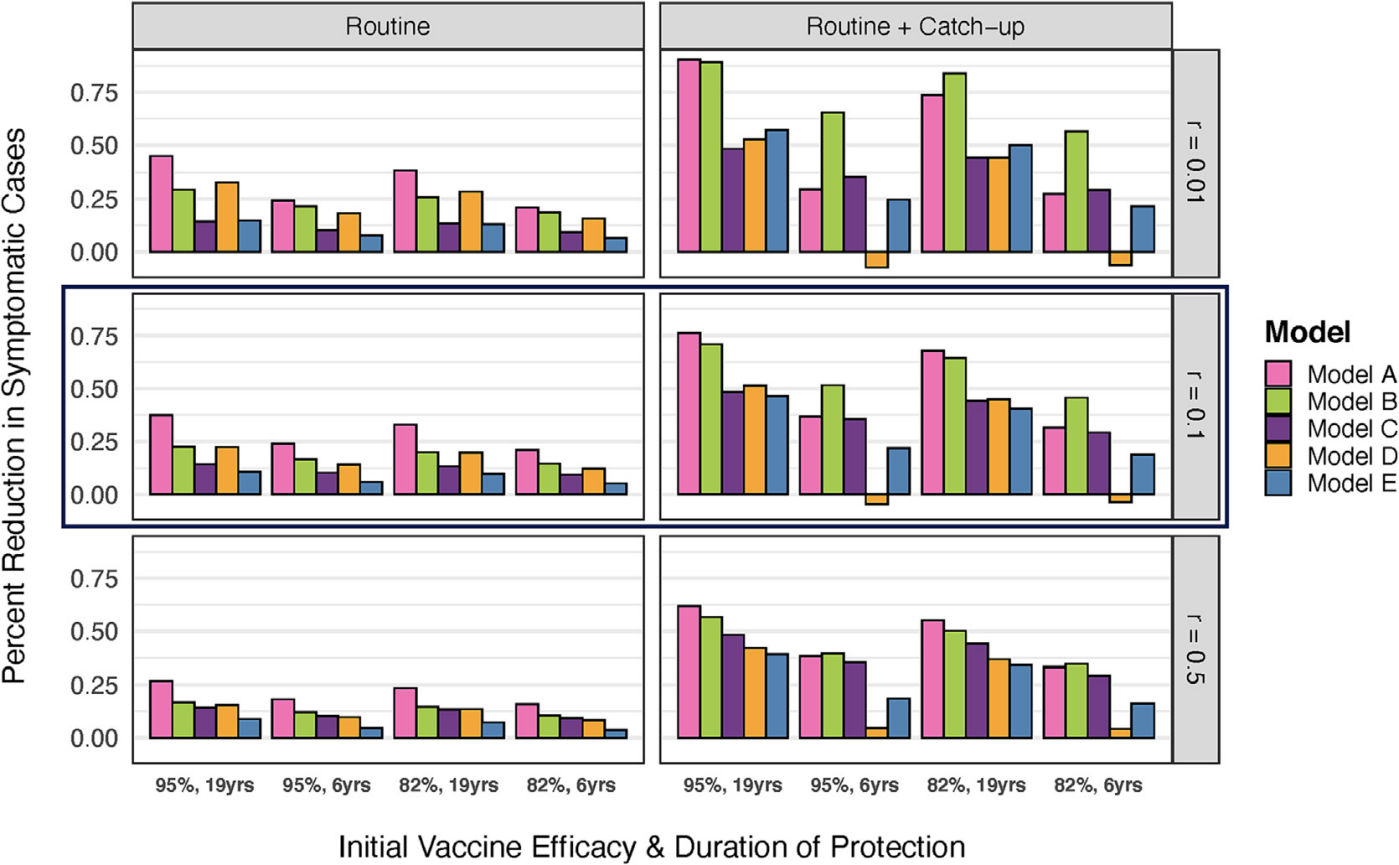
Model predictions for the percent reduction in the incidence of typhoid fever cases over 10 years. We examined two different vaccination strategies (routine or routine plus a catch-up campaign) and three different assumptions for the relative infectiousness of chronic carriers (*r*); the base-case scenario assumes *r* = 0.1 (outlined). Results are shown for each of the five models (colored bars) and for each combination of assumptions regarding the initial vaccine efficacy (95 % or 82 %) and average duration of protection (19 years or 6 years) (horizontal axis groups).

Routine vaccination with TCVs at 9 months of age was predicted to result in a median (range) reduction in the number of symptomatic cases of 17 % (9–45 %) over 10-year time horizon under an optimistic scenario for vaccine efficacy and duration of protection (*VE*_0_ = 95 %, 1/*ω_v_* = 19 years), and an 11 % (4–21 %) reduction under a pessimistic scenario (*VE*_0_ = 82 %, 1/*ω_v_* = 6 years) (Fig. 2). Model A consistently predicted the highest routine vaccine impact for all initial vaccine efficacy and duration of protection scenarios, while the Model E consistently predicted the smallest impact. The Models B and D showed similar predictions for all scenarios, falling in between those of Model A on the high end and Models C and E on the low end. When the relative infectiousness of chronic carriers was 0.5, the predicted impact of routine vaccination was lower and more similar across the five models.

The addition of an initial catch-up campaign among 9-month to < 15-year-olds was generally predicted to lead to further reductions in incidence, ranging from a median (range) reduction of 53 % (39–90 %) in the optimistic scenario and a 29 % (-6–57 %) reduction in the pessimistic scenario. Under the routine plus catch-up campaign strategy, Model A generally predicted the greatest vaccine impact when the average duration of protection was assumed to be 19 years. However, when the average duration of protection was assumed to be 6 years, Model B predicted the greatest impact. Model D predicted a net increase in typhoid cases when the duration of protection was 6 years and the relative infectiousness of chronic carriers was low. Model E generally predicted a lower vaccine impact than Model C, except when the average duration of protection was 19 years and the relative infectiousness of chronic carriers was 0.01.

### 3.2 Trends across time

**Fig. 3:**
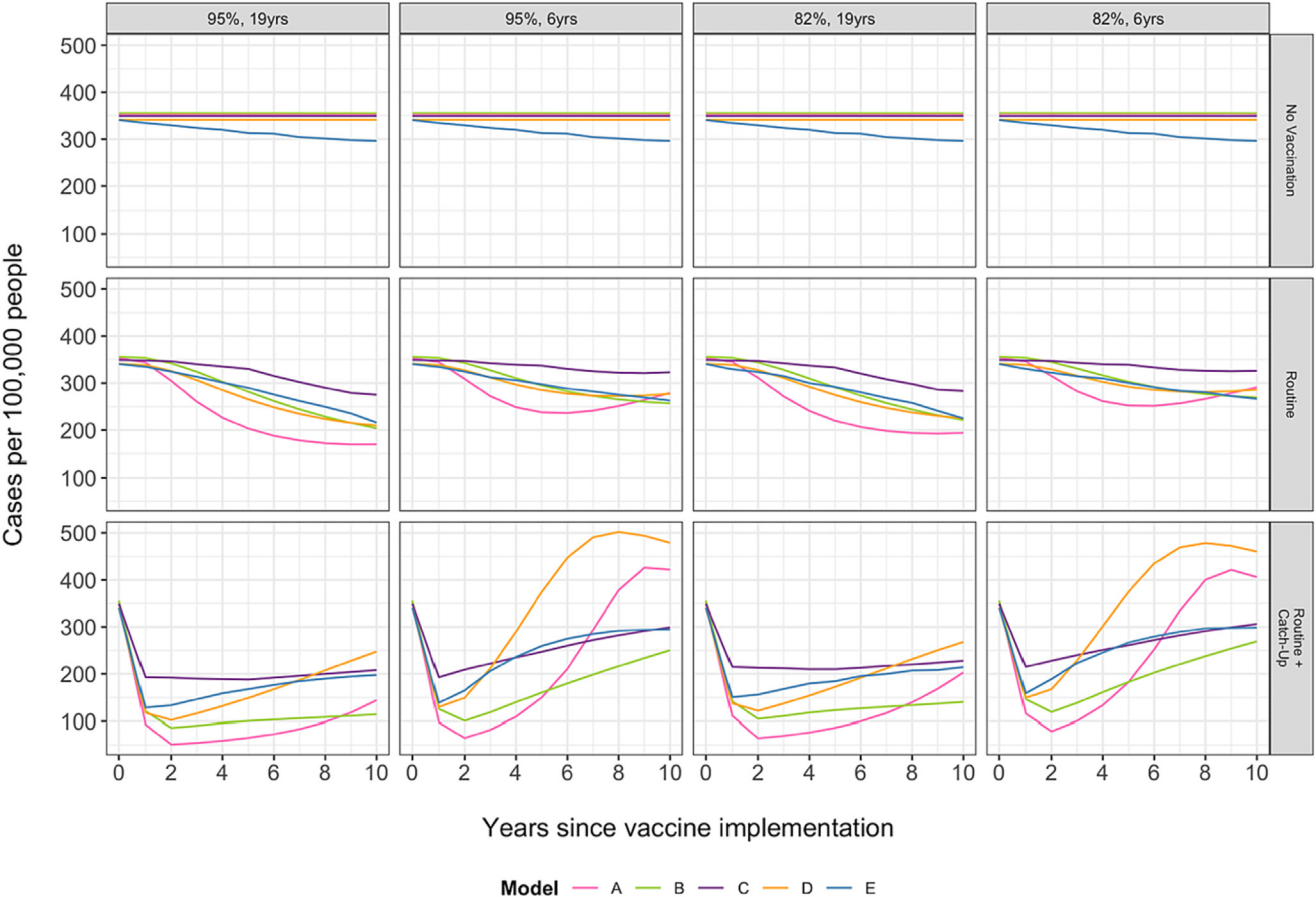
Time series of annual model-predicted cases per 100,000 population for 10 years after vaccine introduction. Projected cases are shown for different assumptions of initial vaccine efficacy (95 % or 82 %) and average duration of protection (19 years or 6 years), and for each vaccination strategy: no vaccination, routine vaccination, and routine vaccination plus a catch-up campaign. Results shown are for the base-case scenario assuming the relative infectious of chronic carriers is 0.1.

The trajectory of projected typhoid cases per 100,000 people varied for the different models and vaccination assumptions. Under the routine vaccination only scenario, when the duration of protection was assumed to be short (6 years), most of the models predicted that the decline in cases would level off towards the end of the 10-year period or begin to rebound (Fig. 3). In particular, Model A predicted a rebound in cases beginning around the 6-year mark, while Model D also predicted a slight increase in cases around year 9. No rebound was predicted when the duration of protection was long (19 years).

All models predicted a marked immediate, short-term impact for routine vaccination plus a catch-up campaign. However, when the duration of protection was short, all models predicted a rebound in cases beginning at the 2-year mark. Models A and D, in particular, predicted an increase in incidence exceeding that of the no vaccination strategy after 5 or more years. When the duration of protection is long, the rebound in incidence was less extreme, but Models A and D still predicted that incidence would be similar to or greater than that projected under routine vaccination alone at 10 years. The other models tended to predict a more stable or modest increase in incidence (compared to the lower post-campaign incidence) over time.

### 3.3 Cost-effectiveness analysis

**Fig. 4:**
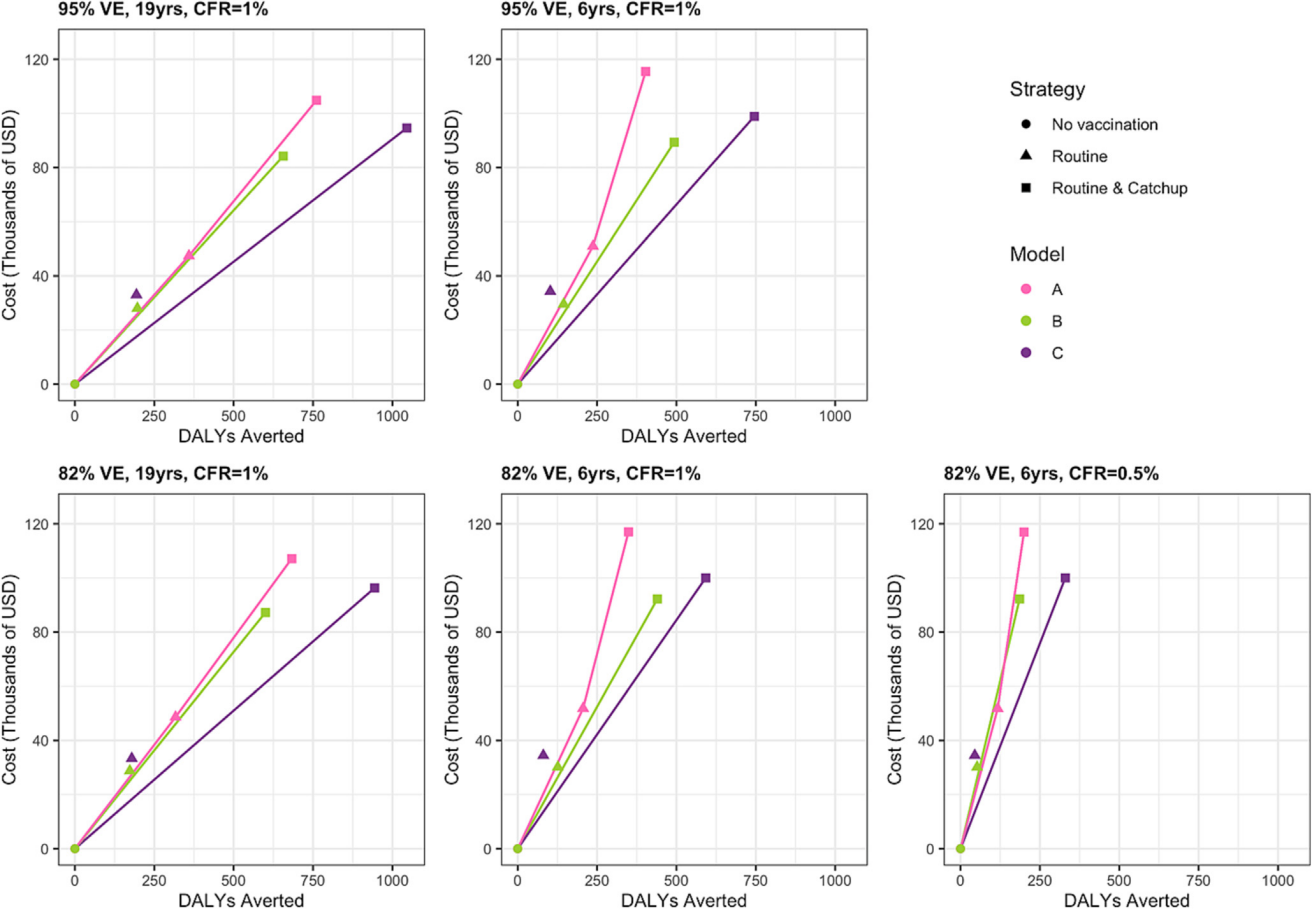
Cost-effectiveness planes. Incremental costs compared to a no vaccination strategy (in thousands of 2016 USD) vs benefits (DALYs averted) for five scenarios of initial vaccine efficacy (VE), average duration of protection (yrs), and case fatality risk (CFR).

Three of the five models – Models A, B, and C – also evaluated the cost-effectiveness of TCV introduction. The routine vaccination only strategy was weakly dominated under all assumptions tested in Models B and C (Table 3). When compared to no vaccination, the ICERs for the routine vaccination plus catch-up campaign strategy for these two models varied from $95 to $381 per DALY averted for the optimistic and pessimistic scenarios, respectively. For Model A, the ICER for routine vaccination compared to no vaccination varied from $132 to $443 per DALY averted, which was lower than the ICER for routine vaccination plus a catch-up campaign compared to routine vaccination only. Model A predicted higher costs and greater benefits for the routine only strategy compared to the other two models (Fig. 4). Model C predicted the greatest benefits for the routine vaccination plus catch-up campaign strategy, with costs between Models A and B. For all vaccination strategies, models, and assumptions of initial vaccine efficacy, duration of protection, and CFR, the ICERs were well below the GDP per capita in India ($1,900 in 2020) [40].

**Table 3:** Symptomatic cases and deaths averted, total and incremental costs and benefits, and incremental cost-effectiveness ratios for routine vaccination and routine vaccination plus catch-up campaign strategies. Results are based on model-predicted vaccine impact results assuming the relative infectiousness of chronic carriers is 0.1. Incremental costs and benefits and ICERs are shown for different assumptions of vaccine efficacy (VE), duration of protection (years), and case fatality rate (CFR).

**Model**	**Strategy**	**Symptomatic cases (deaths) averted**	**Total cost (USD**	**Total benefit (DALYs averted)**	**Incremental cost (USD) versus next best non-dominated alternative**	**Incremental DALYs averted versus next best non-dominated alternative**	**ICER versus next best non-dominated alternative (USD per DALY averted)**
**Scenario 1: 95 % VE; 19yrs; 1 % CFR**							
A	No vaccination	–	28,675	–	–	–	–
	Routine vaccination	1,318 (15)	76,094	359	47,419	359	132
	Routine + campaign vaccination	2,692 (30)	133,530	761	57,436	402	143
B	No vaccination	–	28,028	–	–	–	–
	Routine vaccination	800 (9)	56,059	198	–	–	Weakly dominated
	Routine + campaign vaccination	2,517 (28)	112,254	646	84,227	646	130
C	No vaccination	–	30,325	–	–	–	–
	Routine vaccination	338 (4)	63,304	194	–	–	Weakly dominated
	Routine + campaign vaccination	1,691 (19)	129,547	1,045	99,222	1,045	95
**Scenario 2: 95 % VE; 6yrs; 1 % CFR**							
A	No vaccination	–	28,693	–	–	–	–
	Routine vaccination	852 (9)	79,650	237	50,956	237	215
	Routine + campaign vaccination	1,301 (14)	144,170	403	64,520	166	389
B	No vaccination	–	28,028	–	–	–	–
	Routine vaccination	591 (7)	57,593	147	–	–	Weakly dominated
	Routine + campaign vaccination	1,832 (20)	117,378	484	89,351	484	185
C	No vaccination	–	30,325	–	–	–	–
	Routine vaccination	178 (3)	64,790	103	–	–	Weakly dominated
	Routine + campaign vaccination	1,181 (13)	133,487	745	103,162	745	138
**Scenario 3: 82 % VE; 19yrs; 1 % CFR**							
A	No vaccination	–	28,687	–	–	–	–
	Routine vaccination	1,162 (13)	77,331	317	48,644	317	154
	Routine + campaign vaccination	2,397 (27)	135,845	683	58,515	367	160
B	No vaccination	–	28,028	–	–	–	–
	Routine vaccination	703 (8)	56,784	174	–	–	Weakly dominated
	Routine + campaign vaccination	2,289 (25)	115,289	589	87,262	589	148
C	No vaccination	–	30,325	–	–	–	–
	Routine vaccination	302 (3)	63,577	179	–	–	Weakly dominated
	Routine + campaign vaccination	1,529 (17)	130,880	944	67,303	944	107
**Scenario 4: 82 % VE; 6yrs; 1 % CFR**							
A	No vaccination	–	28,727	–	–	–	–
	Routine vaccination	738 (8)	80,585	206	51,858	206	252
	Routine + campaign vaccination	1,113 (12)	145,759	349	65,174	143	456
B	No vaccination	–	28,028	–	–	–	–
	Routine vaccination	515 (6)	58,158	129	–	–	Weakly dominated
	Routine + campaign vaccination	1,624 (18)	120,269	430	92,241	430	214
C	No vaccination	–	30,325	–	–	–	–
	Routine vaccination	138 (2)	64,790	81	–	–	Weakly dominated
	Routine + campaign vaccination	933 (10)	135,495	592	70,705	592	178
**Scenario 5: 82 % VE; 6yrs; 0.5 % CFR**							
A	No vaccination	–	28,688	–	–	–	–
	Routine vaccination	747 (5)	80,478	117	51,790	117	443
	Routine + campaign vaccination	1,137 (7)	145,661	200	65,184	83	790
B	No vaccination	–	28,028	–	–	–	–
	Routine vaccination	515 (3)	58,158	72	–	–	Weakly dominated
	Routine + campaign vaccination	1,624 (10)	120,269	242	92,241	242	381
C	No vaccination	–	30,325	–	–	–	–
	Routine vaccination	138 (1)	64,790	45	–	–	Weakly dominated
	Routine + campaign vaccination	933 (6)	135,495	330	70,705	330	319

### 3.4 Sensitivity analysis

The predicted vaccine impact generally decreased as the relative infectiousness of chronic carriers (*r*) increased. For example, for routine vaccination only, a median (range) reduction in cases of 18 % (7–45 %) was predicted across all models and combinations of initial vaccine efficacy and duration of protection when *r* = 0.01, while a 13 % (4–27 %) reduction was predicted when *r* = 0.5.

For the Models B and E, vaccine impact and projected annual incidence were similar when vaccine protection was assumed to be “leaky” compared to “all-or-nothing” as we assumed in the primary analysis (Figures S3-S4). The percent reduction in symptomatic cases was predicted to be very similar between the different assumptions for Model B; at most, the predictions differed by 3 %. The results were more disparate for Model E; the “leaky” assumption predicted a vaccine impact up to 22 % lower than “all-or-nothing” (Figure S3).

When baseline incidence was assumed to be 80 % lower, routine vaccination and routine vaccination plus a catch-up campaign were no longer cost-effective at a willingness-to-pay (WTP) threshold equal to India’s per capita GDP for Models A and B under the pessimistic Scenario 5. Furthermore, Model A predicted that routine vaccination alone would be the preferred strategy (i.e. routine vaccination plus a catch-up campaign was no longer cost-effective) under Scenarios 2 and 4 (Table S4).

## 4 Discussion

In countries weighing the cost and benefits of introducing TCVs into the national immunization program, models of vaccine impact and cost-effectiveness can help to inform the decision-making process. Direct model comparisons can help us understand discrepant predictions resulting from differences in model structure and increase confidence in policy when conclusions are consistent across models. Although the magnitude of TCV impact and cost-effectiveness differed between models, we found that all models predicted considerable reductions in typhoid fever incidence following TCV vaccination, particularly when the duration of vaccine-induced immunity is long. Furthermore, all three economic evaluations of TCV introduction found that routine vaccination with or without a catch-up campaign is likely to be cost-effective compared to no vaccination in high-incidence settings such as Kolkata. However, all models predicted that typhoid incidence would remain high, suggesting that additional measures may be needed for typhoid control.

The introduction of routine TCV vaccination alone was predicted to have a modest to moderate impact on typhoid fever incidence. In general, Model A predicted the greatest impact of routine vaccination, while Model E predicted the lowest vaccine impact. This finding can be explained by two key differences in model structure. First, Model E is a stochastic individual-based model, whereas the other dynamic models were deterministic compartmental models. As a result, there were differences in the underlying population demography between the models. While we harmonized the models by assuming a fixed birth rate and initial age distribution, this led to declines in the target population for TCV in the individual-based simulation and more conservative estimates of vaccine impact for this model. Model E predicted typhoid fever incidence would decline even in the absence of vaccination; therefore, the relative benefit of TCV introduction was smaller. Second, Model A differs from the other dynamic models in that it assumes that chronic carriage lasts for 10 years on average, whereas the other models assume chronic carriage is lifelong. Thus, the prevalence of chronic carriers is lower in Model A and, compared to the dynamic other models, they are predicted to play a lesser role than acute cases in driving typhoid transmission (Figure S5), resulting in greater indirect protection (since vaccination prevents acute cases but has little impact on chronic carriers). In assuming that chronic carriers are infectious for life, the other dynamic models provide more conservative estimates of vaccine impact due to lower indirect protection, particularly for Model E, which had the greatest prevalence of chronic carriers (Figure S5) as well as a greater prevalence of infection overall (as indicated by the fitted proportion of infections that are symptomatic; Table S3).

The median model-predicted vaccine impact increased by 36 % with the addition of a catch-up campaign under an optimistic scenario and by 18 % under a pessimistic scenario regarding vaccine efficacy and duration of protection. As with routine vaccination, there were variations in model predictions due to differences in model structure and assumptions. Model A generally predicted greater vaccine impact on typhoid incidence than the other models when the average duration of protection was long; however, Model B predicted greater impact than the other models when the average duration was short. This is related to the pronounced rebound in cases predicted by Model A following the initial post-campaign decline in incidence. While indirect protection is greatest for Model A (driven in part by assumptions about the duration of carriage), this can yield a build-up of susceptible individuals, thus only delaying the time to infection when the duration of vaccine-induced immunity is short. Model B predicts lower indirect protection due to the prevalence of subclinical infections and a more gradual rebound in incidence following a catch-up campaign. Model D predicted the largest rebound in typhoid fever incidence following routine vaccination plus a catch-up campaign when the duration of vaccine-induced immunity is short, potentially leading to a negative vaccine impact (i.e., increase in total typhoid fever cases over 10 years following vaccine introduction). Model D estimates that immunity following typhoid infection is longer than when the exponentially-distributed average vaccine-induced immunity is assumed to be 6 years, and does not differentiate between vaccine-induced immunity among those who are fully susceptible versus those who have experienced a previous typhoid infection. Therefore, vaccination could perversely lead to a shorter duration of immunity among those who were previously infected, possibly explaining the large rebound in incidence following the catch-up campaign and the negative predicted vaccine impact for this model. We believe this to be an artifact of this model’s structure and parameterization.

Our analysis further highlights that understanding the role chronic carriers play in typhoid transmission is key to predicting TCV impact. However, efforts to assess the prevalence of chronic carriage in typhoid-endemic populations have proven challenging [41]. Some recent evidence suggests carriers play a minor role in typhoid transmission, but in general both the duration and infectiousness of chronic carriage is uncertain [41], [42]. Due to the administration of antibiotics for various conditions throughout a lifetime, the resolution of the carrier state through antibiotic use is plausible [43]. However, vaccination of chronic carriers is unlikely to cure their infection, since chronic carriers typically already have very high levels of Vi antibodies [44], [45], [46], [47]. We found that as the relative infectiousness of chronic carriers increased, the vaccine impact predicted by the dynamic models decreased. This finding is consistent with previous typhoid modeling studies and can be explained by the decreased indirect protection afforded by vaccination when chronic carriers account for a greater share of overall typhoid transmission [32].

Importantly, we also demonstrate that a static model does not necessarily provide conservative estimates of vaccine impact on typhoid fever. Since Model C does not account for indirect protection (i.e., the protection afforded to individuals who did not receive the vaccine in a population where others have been vaccinated), we expected it to predict a lower vaccine impact than the dynamic models. However, it often predicted a greater impact than Model E and occasionally Model D, particularly for the routine plus catch-up campaign strategy. Rebounds in typhoid fever transmission following the waning of vaccine-induced immunity, as well as trends in typhoid incidence in the absence of vaccination, are not fully accounted for by the static model, making its predictions non-conservative, at least for the 10-year time horizon considered. Interestingly, Model C also predicted a greater benefit of routine vaccination plus a catch-up campaign in terms of DALYs averted compared to Model B. As a result, the ICER of routine vaccination plus a catch-up campaign ends up being *lowest* for Model C under both optimistic and pessimistic scenarios regarding the vaccine effectiveness, even though dynamic Models A and B predicted a greater vaccine impact.

GDP per capita is a commonly-used WTP threshold against which to benchmark cost-effectiveness estimates. All three models included in the economic evaluation suggested that routine vaccination with TCVs with or without a catch-up campaign would be cost-effective in Kolkata even at a WTP threshold of 50 % of the GDP per capita of India. For Models B and C, the routine plus catch-up campaign strategy was predicted to have a lower ICER than the routine only strategy, and would be cost-effective even at a WTP threshold of 25 % of the GDP per capita of India. However, for Model A, the routine vaccination only strategy had a lower ICER than the strategy including a catch-up campaign. Thus, when the WTP threshold is reduced to 25 % of GDP per capita in India, the addition of the catch-up campaign is no longer cost-effective under a pessimistic scenario of 82 % initial vaccine efficacy, 6-year average duration of protection, and a 0.5 % CFR. If we assume a lower typhoid fever incidence, neither vaccination strategy would be cost-effective at a WTP threshold equal to the GDP per capita according to Models A and B under the pessimistic scenario. By using common assumptions regarding the severity of illness, treatment seeking, and costs across all three models, we show that when policy implications differ, these differences are driven entirely by our uncertainty in typhoid transmission, immunity, and carriage.

Our results support other analyses predicting the cost-effectiveness of TCV introduction in India. Using a dynamic compartmental model, Ryckman et al. (2021) assessed the cost-effectiveness of different TCV vaccination strategies in India across a 10-year time horizon [48]. Similar to our study, they predicted that, from a healthcare perspective, a routine vaccination plus catch-up campaign strategy would be cost-effective or cost-saving. From a societal perspective, they projected that all strategies tested would be cost-saving. They also found that vaccination was more cost-effective in urban areas, which tend to have higher incidence. Chauhan et al. (2021) used a static decision-analytic model, most similar to Model C in our study, to predict the cost-effectiveness of TCV introduction over a 15-year period [49]. Testing a routine vaccination strategy at a WTP threshold of around $1900 USD (the threshold used in our study), they predicted that TCV use in urban settings would be cost-effective when indirect costs were excluded (similar to our study) and cost-saving when indirect costs were included (i.e., from a societal perspective). Taken together, the findings of these three studies suggest that TCV introduction is likely to be cost-effective in India, particularly in high-incidence urban centers.

Although we explored a variety of different scenarios for the initial vaccine efficacy, duration of vaccine-induced immunity, and relative infectiousness of chronic carriers, we did not thoroughly explore the parameter uncertainty for the various models. However, this has been done in individual modeling studies [15], [32], [34], [50]. In addition, we only explored model predictions of vaccine impact and cost-effectiveness for a single high-burden setting (chosen for the availability of data on incidence and cost of illness). Differences in model structure may be more influential in medium-burden settings, as illustrated by our sensitivity analysis. However, the sensitivity analysis is an oversimplification, since we assumed the same reduction in typhoid fever incidence across all age groups. In reality, the average age of typhoid fever cases tends to be higher in lower incidence settings [1]. Further work is needed to predict the impact and cost-effectiveness of TCV introduction in settings with different baseline levels of typhoid fever burden as the policy recommendations may differ.

Overall, our comparison of models of typhoid transmission and vaccination led to a unanimous finding that TCV introduction is predicted to be impactful and cost-effective in a high-burden setting such as Kolkata. The study also allowed us to examine the impact of differences in model structure on projections of vaccine impact and cost-effectiveness. These differences reflect gaps in our understanding of the natural history of disease, and can lead to substantial differences in predicted vaccine impact. Parameters that strongly influenced model projections included the role of chronic carriers in transmission, the characteristics of the vaccine (duration of protection, initial vaccine efficacy), and, for the economic evaluation, the case fatality risk. We also found that static models of typhoid vaccine impact do not necessarily provide conservative estimates for the cost-effectiveness of TCVs. Importantly, despite large differences in the predicted impact and cost-effectiveness of TCVs, their introduction is likely to be cost-effective in a high-burden setting. However, typhoid fever burden would still remain high, suggesting that vaccination alone would be insufficient for control and should be implemented alongside improvements in water, sanitation, and hygiene.

## Funding source

This work was supported, in whole or in part, by the Bill & Melinda Gates Foundation, via TyVAC [OPP1151153] and the Vaccine Impact Modelling Consortium [Grant Number INV-009125]. NCL is supported by the National Institutes of Health, NIAID New Innovator Award (DP2 AI170485). The views expressed are those of the authors and not necessarily those of the Consortium or its funders. The funder had no role in the identification, design, conduct, or reporting of the analyses. Under the grant conditions of the Foundation, a Creative Commons Attribution 4.0 Generic License has already been assigned to the Author Accepted Manuscript version that might arise from this submission.

## CRediT authorship contribution statement

**Holly Burrows:** investigation, data curation, formal analysis, visualization, writing - original draft, writing - review & editing. **Marina Antillón:** conceptualization, investigation, methodology, software, writing - review & editing. **Jillian S. Gauld:** conceptualization, investigation, methodology, software, writing - review & editing. **Jong-Hoon Kim:** conceptualization, investigation, methodology, software, writing - review & editing. **Vittal Mogasale:** investigation, methodology, software, writing - review & editing. **Theresa Ryckman:** investigation, methodology, software, writing - review & editing. **Jason R. Andrews:** conceptualization, investigation, methodology, software, writing - review & editing. **Nathan C. Lo:** conceptualization, investigation, methodology, software, writing - original draft, writing - review & editing, supervision, funding acquisition.

## Declaration of Competing Interest

The authors declare the following financial interests/personal relationships which may be considered as potential competing interests: VEP is a member of the WHO Immunization and Vaccine-related Implementation Research Advisory Committee (IVIR-AC). All other authors have no conflicts to declare.

## Data Availability

Our study uses published data, which can be found in Table S1
