# Supplementary material for: Comparison of model predictions of typhoid conjugate vaccine public health impact and cost-effectiveness
Source: Vaccine. 2023 Jan 23;41(4):965–75. doi: 10.1016/j.vaccine.2022.12.032 (PMC9880559; doi:10.1016/j.vaccine.2022.12.032)
Supplement: Supplementary data 1 [file mmc1.docx]

**Supplementary Tables and Figures**

Holly Burrows^a^, Marina Antillón^a,b,c^, Jillian S. Gauld^d^, Jong-Hoon Kim^e^, Vittal Mogasale^f^, Theresa Ryckman^g^, Jason R. Andrews^h^, Nathan C. Lo^i^, Virginia E. Pitzer^a*^

*^a^ Yale School of Public Health, Yale University, New Haven, Connecticut, USA*

*^b^ Swiss Tropical and Public Health Institute, Allschwil , Switzerland*

*^c^ University of Basel, Basel, Switzerland*

*^d^ Institute for Disease Modeling, Bill & Melinda Gates Foundation, Seattle, Washington, USA*

*^e^ Public Health, Access, and Vaccine Epidemiology (PAVE) Unit, International Vaccine Institute, Seoul, Korea*

*^f^ Policy and Economic Research Department, International Vaccine Institute, Seoul, 08826, Republic of Korea (Current affiliation: Department of Health Systems Governance and Financing, World Health Organization, Geneva, Switzerland.)*

*^g^ Department of Epidemiology, Johns Hopkins Bloomberg School of Public Health, Baltimore, MD, USA*

*^h^ Division of Infectious Diseases and Geographic Medicine, Stanford University School of Medicine, Stanford, CA, USA*

*^i^ Division of HIV, Infectious Diseases, and Global Medicine, University of California, San Francisco, San Francisco, California, USA*

**Table S1.** Age-specific incidence of typhoid fever cases during a one-year active surveillance study in Kolkata, India, and other age-specific model parameters [1].

| **Age group** | **Number of confirmed typhoid cases** | **Person-years of observation** | **Typhoid incidence (per 100K person-yrs)** | **Fraction of population** | **Death rate (per person per year)** | **Prob of becoming a chronic carrier** |
| --- | --- | --- | --- | --- | --- | --- |
| <2 yr | 1 | 1488 | 67.2 | 0.025 | 0.0059 | 0.003 |
| 2-4 yr | 7 | 3102 | 225.7 | 0.051 | 0.0024 | 0.003 |
| 5-9 yr | 28 | 5609 | 499.2 | 0.093 | 0.001 | 0.003 |
| 10-19 yr | 39 | 12949 | 301.2 | 0.214 | 0.0011 | 0.003 |
| 20-29 yr | 12 | 12049 | 99.6 | 0.199 | 0.0016 | 0.021 |
| 30-39 yr | 4 | 9833 | 40.7 | 0.163 | 0.0026 | 0.044 |
| 40-49 yr | 3 | 6994 | 42.9 | 0.116 | 0.0052 | 0.088 |
| 50+ yr^*^ | 1 | 8448 | 11.8 | 0.14 | 0.0479 | 0.092 |
| All ages | ***95*** | ***60472*** | ***157.1*** |  |  |  |

^*^Models assume a maximum age of 65 years to maintain an approximately stable age distribution.

**Table S2.** Cost-effectiveness analysis assumptions for previous models and common assumptions for model comparison.

| **Parameter definition** | **Common Value** | **Source** |
| --- | --- | --- |
| *Treatment seeking (as a proportion of symptomatic cases)* | | |
| No treatment  Outpatient treatment  Inpatient treatment | 0.25  0.69 (=0.75*0.92)  0.06 (=0.75*0.08) | Assumption; [2] |
| *Treatment outcomes (probability)* | | |
| IP/other complication (for inpatients)  Death, no treatment  Death, outpatient treatment  Death, inpatient treatment (uncompl)  Death, inpatient treatment (IP/other) | 0.18  0.01  0.01  0.01  0.11 | Assumption; [3]; [4] (based on estimate for IP in Asia) |
| *Treatment costs (2016 US$)* |  |  |
| Outpatient  Inpatient | $2.52  $125.00 | [5] (adjusted for inflation) |
| ***DALY estimation*** | | |
| *Probability of health states*  Acute infectious disease, mild  Acute infectious disease, moderate  Acute infectious disease, severe  Intestinal perforation & related | 0.125 (=0.5*0.25)  0.353  0.511  0.011 (=0.06*0.18) | Based on assumption that 50% of those not seeking care have mild (vs moderate) illness and 33% of outpatients have moderate (vs severe) illness |
| *DALY weight*  Acute infectious disease, mild  Acute infectious disease, moderate  Acute infectious disease, severe  Intestinal perforation & related | 0.005  0.053  0.210  0.326 | [6] (DALY weights from 2010 GBD were used because countries where interviews were conducted were more representative of LMICs) |
| *Average DALY weight* | 0.131 [7] |  |
| Duration of illness | 16 days | [1, 7, 8] |
| Life expectancy | 65 years | [9] |
| ***Vaccination costs (2016 USD)*** | | |
| Procurement cost (per dose) | $2.70 | Assumption; based on current price of Bharat ViPS vaccine |
| Injection and safety equipment | $0.23 | [10] (based on Gavi forecast) |
| Delivery cost, routine | $0.89 | [11-13] |
| Delivery cost, campaign | $0.42 | [13, 14] |

**Table S3.** Mean (95% CI) estimated parameters for the transmission dynamic models, when the relative infectiousness of chronic carriers is 0.01, 0.1, and 0.5 (from top to bottom).

| **Parameter Definition** | **Model A** | **Model B** | **Model D** | **Model E*** |
| --- | --- | --- | --- | --- |
| Basic reproductive number (R0) | NA | 2.54 (2.22, 2.86)  5.43 (4.52, 6.34)  13.16 (9.54, 16.79) | 6.48 (6.24, 7.22)  10.70 (9.56 12.01)  17.30 (15.15, 19.82) | NA |
| Annual rate of waning immunity (to reinfection resulting in *clinical* disease) | 5.95 (2.03, 7.70) x10^-4^  9.41 (3.82, 13.1) x10^-4^  4.12 (0.08, 9.12) x10^-4^ | NA | 3.46 (0.140, 13.1) x 10^-4^  3.53 (0.140, 13.9) x 10^-4^  3.60 (0.150, 14.4) x 10^-4^ | Duration of immunity: 22750 |
| Proportion of infections that are *symptomatic* | 0.176 (0.172, 0.186)  0.174 (0.166, 0.178)  0.175 (0.166, 0.184) | 0.20 (0.16, 0.25)  0.20 (0.16, 0.25)  0.20 (0.16, 0.24) | NA | 0.06  0.046  0.46 |
| Proportion of infections that are *diagnosed* | NA | NA | 0.0851 (0.0794, 0.0907)  0.113 (0.106, 0.121)  0.113 (0.106, 0.121) | NA |
| Relative risk of infection for <2 yr olds | NA | 0.13 (0.03, 0.43)  0.13 (0.03, 0.42)  0.12 (0.03, 0.42) | 0.0718 (0.0549, 0.0928)  0.0716 (0.0546, 0.0920)  0.0714 (0.0542, 0.0919) | NA |
| Relative risk of infection for 2-4 yr olds | NA | 0.28 (0.12, 0.53)  0.28 (0.12, 0.53)  0.27 (0.11, 0.53) | 0.270 (0.227, 0.319)  0.270 (0.228, 0.228)  0.270 (0.226, 0.318) | NA |
| Relative risk of infection of <5 yr olds | 0.175 (0.150, 0.185)  0.162 (0.139, 0.192)  0.175 (0.140, 0.182) | NA | NA | NA |
| Rate of transition from “susceptible to subclinical reinfection (S1)” to “fully susceptible (S2)” | NA | 0.42 (0.42, 0.42) x 10^^-13^  1.07 (1.07, 1.07) x 10^^-13^  1.04 (1.04, 1.04) x 10^^-13^ | NA | NA |
| Short-cycle (person-to-person) transmission coefficient | 1.92 (1.70, 2.64)  1.48 (1.39, 2.18)  1.73 (1.56, 2.17) | NA | NA | NA |
| Long-cycle (water-borne) transmission coefficient | 3.97 (3.11, 4.54)  4.07 (3.19, 4.21)  1.43 (0.96, 1.91) | NA | NA | NA |
| Long-cycle exposure rate | NA | NA | NA | 0.53  0.43  0.25 |
| Short-cycle exposure rate | NA | NA | NA | 1  0.84  0.44 |
| Reduction in susceptibility after clinical or sub-clinical infection | NA | NA | NA | 0.79 |
| Susceptible introduction curve | NA | NA | NA | -0.70 |
| Age at full susceptibility (days) | NA | NA | NA | 3865 |

*Reduction in susceptibility after infection, susceptible introduction curve, age at full susceptibility, and duration of immunity were fitted for relative infectiousness of chronic carriers=0.1 and held constant for relative infectiousness = 0.01 and 0.05.

**Table S4.** Results of the sensitivity analysis for the cost-effectiveness analysis, where incidence was assumed to be 80% lower than observed in Kolkata. Symptomatic cases and deaths averted, total and incremental costs and benefits, and incremental cost-effectiveness ratios for routine vaccination and routine vaccination plus catch-up campaign strategies are shown. Results are based on model-predicted vaccine impact results assuming the relative infectiousness of chronic carriers is 0.1. Incremental costs and benefits and ICERs are shown for different assumptions of vaccine efficacy (VE), duration of protection (years), and case fatality rate (CFR).

| **Model** | **Strategy** | **Symptomatic cases (deaths) averted** | **Total cost (USD** | **Total benefit (DALYs averted)** | **Incremental cost (USD) versus next best non-dominated alternative** | **Incremental DALYs averted versus next best non-dominated alternative** | **ICER versus next best non-dominated alternative (USD per DALY averted)** |
| --- | --- | --- | --- | --- | --- | --- | --- |
| **Scenario 1: 95% VE; 19yrs; 1% CFR** | | | | | | | |
| A | No vaccination | - | 5,735 | - | - | - | - |
|  | Routine vaccination | 264 (3) | 61,412 | 72 | 55,677 | 72 | 775 |
|  | Routine + campaign vaccination | 538 (6) | 128,185 | 152 | 66,773 | 80 | 380 |
| B | No vaccination | - | 28,028 | - | - | - | - |
|  | Routine vaccination | 160 (2) | 38,413 | 40 | - | - | Weakly dominated |
|  | Routine + campaign vaccination | 503 (6) | 105,725 | 129 | 100,120 | 129 | 774 |
| C | No vaccination | - | 6,064 | - | - | - | - |
|  | Routine vaccination | 67 (1) | 41,080 | 39 | - | - | Weakly dominated |
|  | Routine + campaign vaccination | 338 (4) | 116,267 | 209 | 110,202 | 209 | 527 |
| **Scenario 2: 95% VE; 6yrs; 1% CFR** | | | | | | | |
| A | No vaccination | - | 5,739 | - | - | - | - |
|  | Routine vaccination | 170 (2) | 62,123 | 47 | 56,385 | 47 | 1,187 |
|  | Routine + campaign vaccination | 260 (3) | 130,378 | 81 | 68,255 | 33 | 2,055 |
| B | No vaccination | - | 5,606 | - | - | - | - |
|  | Routine vaccination | 118 (1) | 38,719 | 29 | 33,114 | 29 | 1,124 |
|  | Routine + campaign vaccination | 366 (4) | 106,760 | 97 | 68,040 | 53 | 1,294 |
| C | No vaccination | - | 6,064 | - | - | - | - |
|  | Routine vaccination | 36 (1) | 41,320 | 21 | - | - | Weakly dominated |
|  | Routine + campaign vaccination | 236 (3) | 117,055 | 149 | 110,990 | 149 | 745 |
| **Scenario 3: 82% VE; 19yrs; 1% CFR** | | | | | | | |
| A | No vaccination | - | 5,737 | - | - | - | - |
|  | Routine vaccination | 232 (3) | 61,659 | 63 | 55,922 | 63 | 882 |
|  | Routine + campaign vaccination | 479 (5) | 128,655 | 137 | 66,996 | 73 | 914 |
| B | No vaccination | - | 5,606 | - | - | - | - |
|  | Routine vaccination | 141 (2) | 38,558 | 35 | - | - | Weakly dominated |
|  | Routine + campaign vaccination | 458 (5) | 107,335 | 118 | 101,729 | 148 | 863 |
| C | No vaccination | - | 6,064 | - | - | - | - |
|  | Routine vaccination | 60 (1) | 41,135 | 36 | - | - | Weakly dominated |
|  | Routine + campaign vaccination | 306 (3) | 116,534 | 189 | 110,469 | 189 | 585 |
| **Scenario 4: 82% VE; 6yrs; 1% CFR** | | | | | | | |
| A | No vaccination | - | 5,745 | - | - | - | - |
|  | Routine vaccination | 148 (2) | 62,310 | 41 | 56,565 | 41 | 1,373 |
|  | Routine + campaign vaccination | 223 (2) | 130,688 | 70 | 68,378 | 29 | 2,391 |
| B | No vaccination | - | 5,606 | - | - | - | - |
|  | Routine vaccination | 103 (1) | 38,832 | 129 | - | - | Weakly dominated |
|  | Routine + campaign vaccination | 325 (4) | 108,339 | 430 | 102,733 | 86 | 1,193 |
| C | No vaccination | - | 6,064 | 0 | - | - | - |
|  | Routine vaccination | 28 (0) | 41,377 | 16 | - | - | Weakly dominated |
|  | Routine + campaign vaccination | 187 (2) | 117,457 | 118 | 111,392 | 118 | 940 |
| **Scenario 5: 82% VE; 6yrs; 0.5% CFR** | | | | | | | |
| A | No vaccination | - | 5,738 | - | - | - | - |
|  | Routine vaccination | 149 (1) | 62,289 | 23 | 56,551 | 23 | 2,416 |
|  | Routine + campaign vaccination | 277 (1) | 130,774 | 40 | 68,485 | 17 | 4,147 |
| B | No vaccination | - | 5,606 |  |  |  |  |
|  | Routine vaccination | 103 (1) | 38,832 | 14 | - | - | Weakly dominated |
|  | Routine + campaign vaccination | 325 (2) | 108,339 | 48 | 102,733 | 48 | 2,123 |
| C | No vaccination | - | 6,064 | 0 | - | - | - |
|  | Routine vaccination | 28 (0) | 41,377 | 9 | - | - | Weakly dominated |
|  | Routine + campaign vaccination | 187 (1) | 117,457 | 66 | 111,392 | 66 | 1,334 |


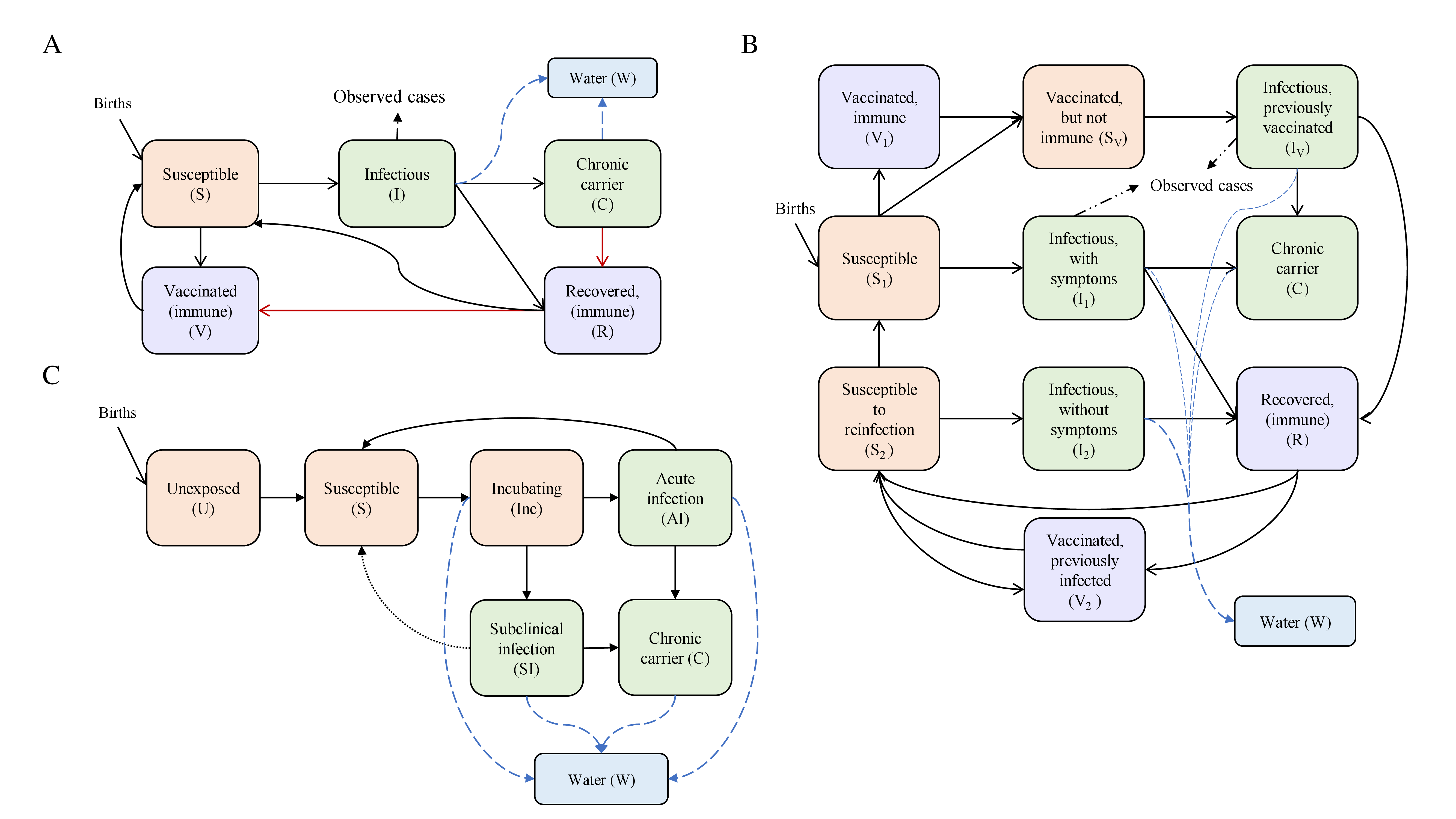


**Figure S1. Compartmental model diagrams detailing model structures**. (A) Models A and D, (B) Model B, (C) Model E. Model A includes the red arrows while D does not. Black dashed arrows indicate compartments contributing to observed cases. Blue dashed lines indicate compartments/states that contribute to the environmental reservoir (W). In Model A, because the modeled duration of immunity from previous infection is longer than the duration of immunity from vaccination, those in the R compartment stay in the R compartment upon vaccination; however, the cost-effectiveness analysis still considers the cost of vaccinating these individuals

**
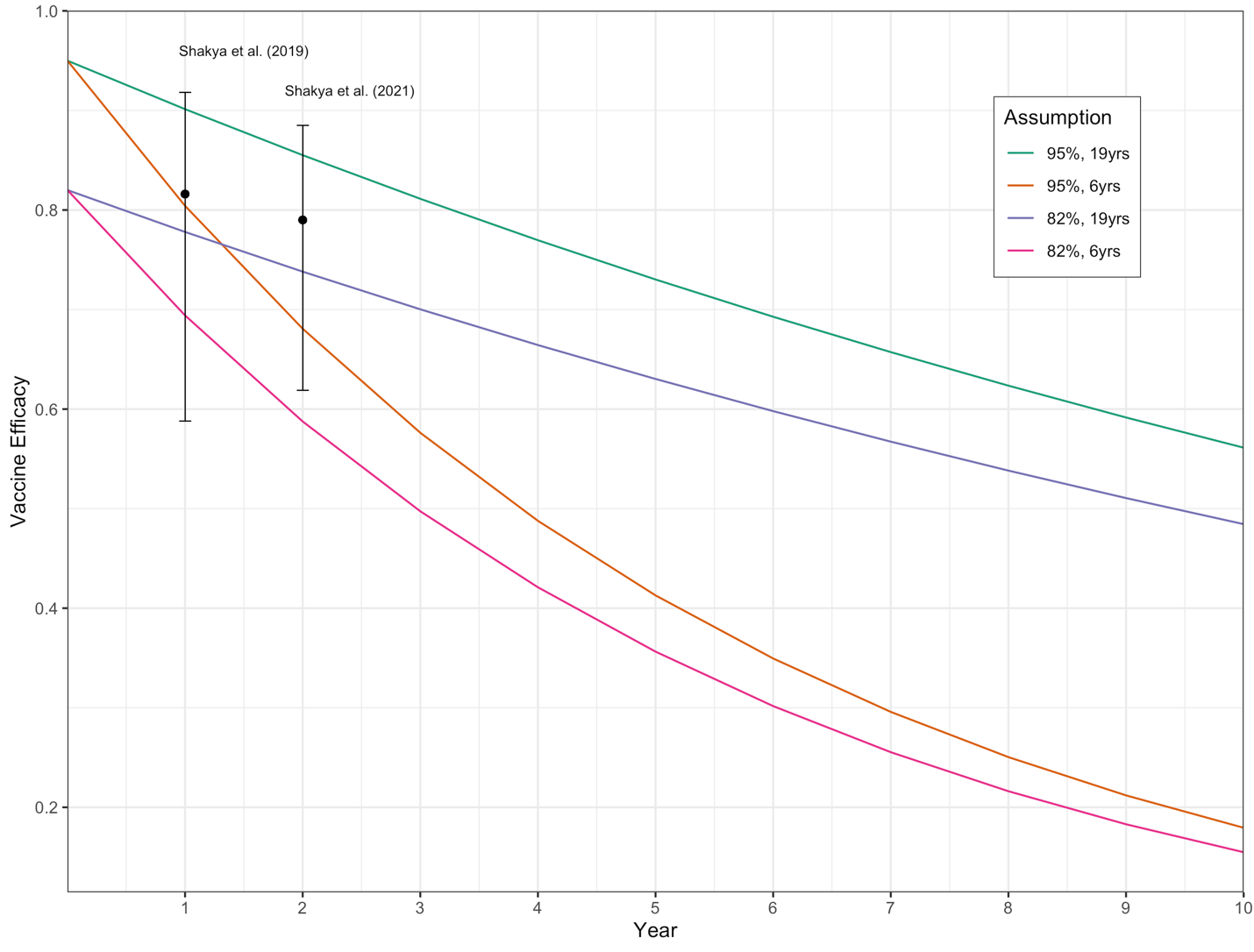
**

**Figure S2. Assumed vaccine efficacy over time for each of the four combinations of assumptions of initial efficacy (*VE*_0_ = 95% or 82%) and duration of protection (1/*ω_v_* = 19 years or 6 years).** Point estimates and 95% confidence intervals of vaccine efficacy in recent trials are shown in black [15, 16].


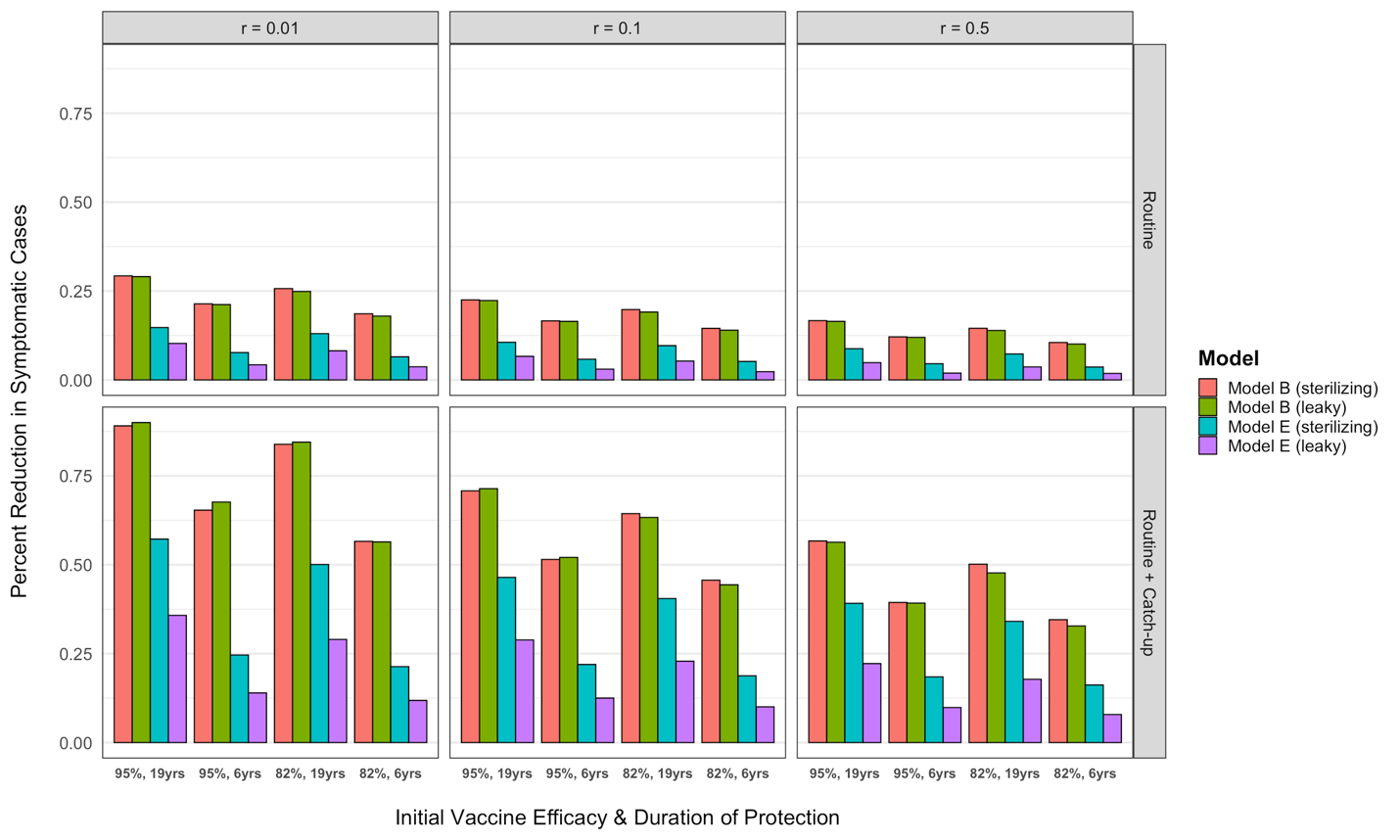


**Figure S3.** Sensitivity analysis for the percent reduction in the incidence of typhoid fever cases, comparing a “leaky” vaccine to an “all-or-nothing” vaccine (as in the primary analysis). Results are shown for six combinations of vaccination strategy (routine or routine plus catch-up campaign) and the relative infectiousness of chronic carriers (r), and for each combination of vaccine efficacy (95% or 82%) and duration of protection (19 years or 6 years) assumptions.

**
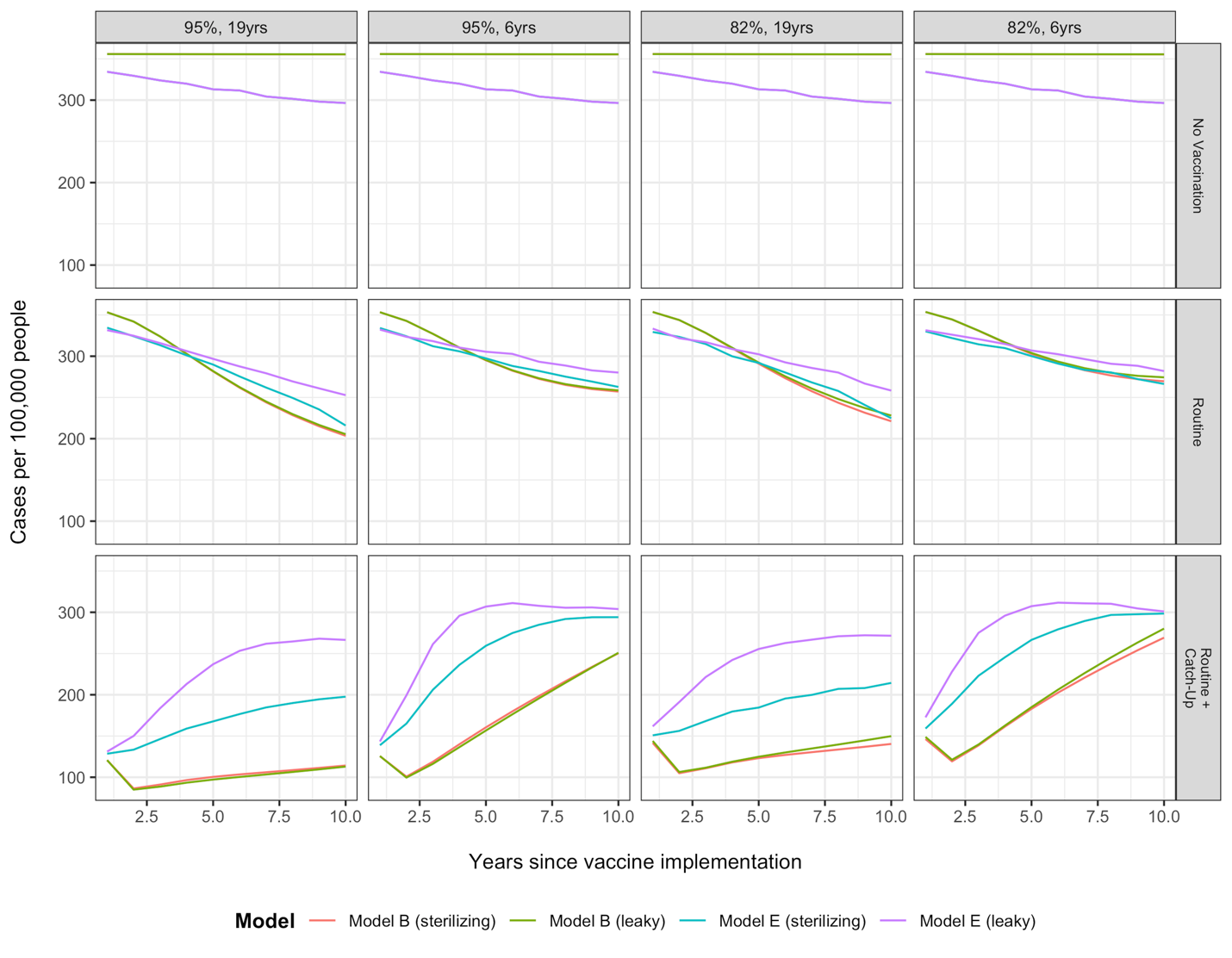
**

**Figure S4.** Sensitivity analysis for the time series of annual model-predicted cases per 100,000 population for 10 years after vaccine introduction, comparing “leaky” and “all-or-nothing” vaccine assumptions. Projected cases are shown for different assumptions of initial vaccine efficacy (95% or 82%) and duration of protection (19 years or 6 years), and for each vaccination strategy.

**
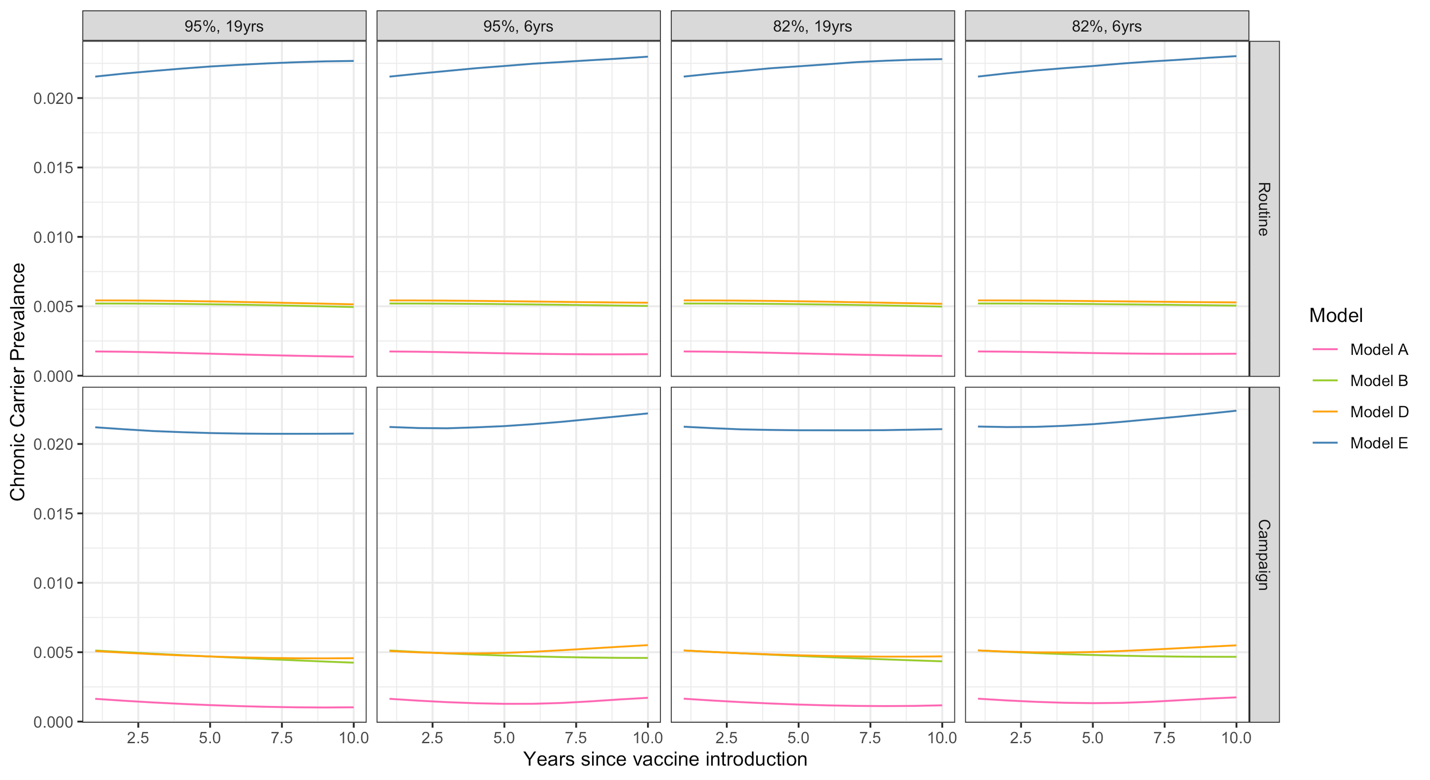
**

**Figure S5. Dynamic model predictions for the prevalence of chronic carriers over 10 years after vaccine introduction.** The projected prevalence is shown for different combinations of initial vaccine efficacy (95% or 82%) and average duration of protection (19 years or 6 years), and for two vaccination strategies (routine and routine plus catch-up campaign). Results are shown for the base-case scenario assuming the relative infectiousness of chronic carriers is 0.1.


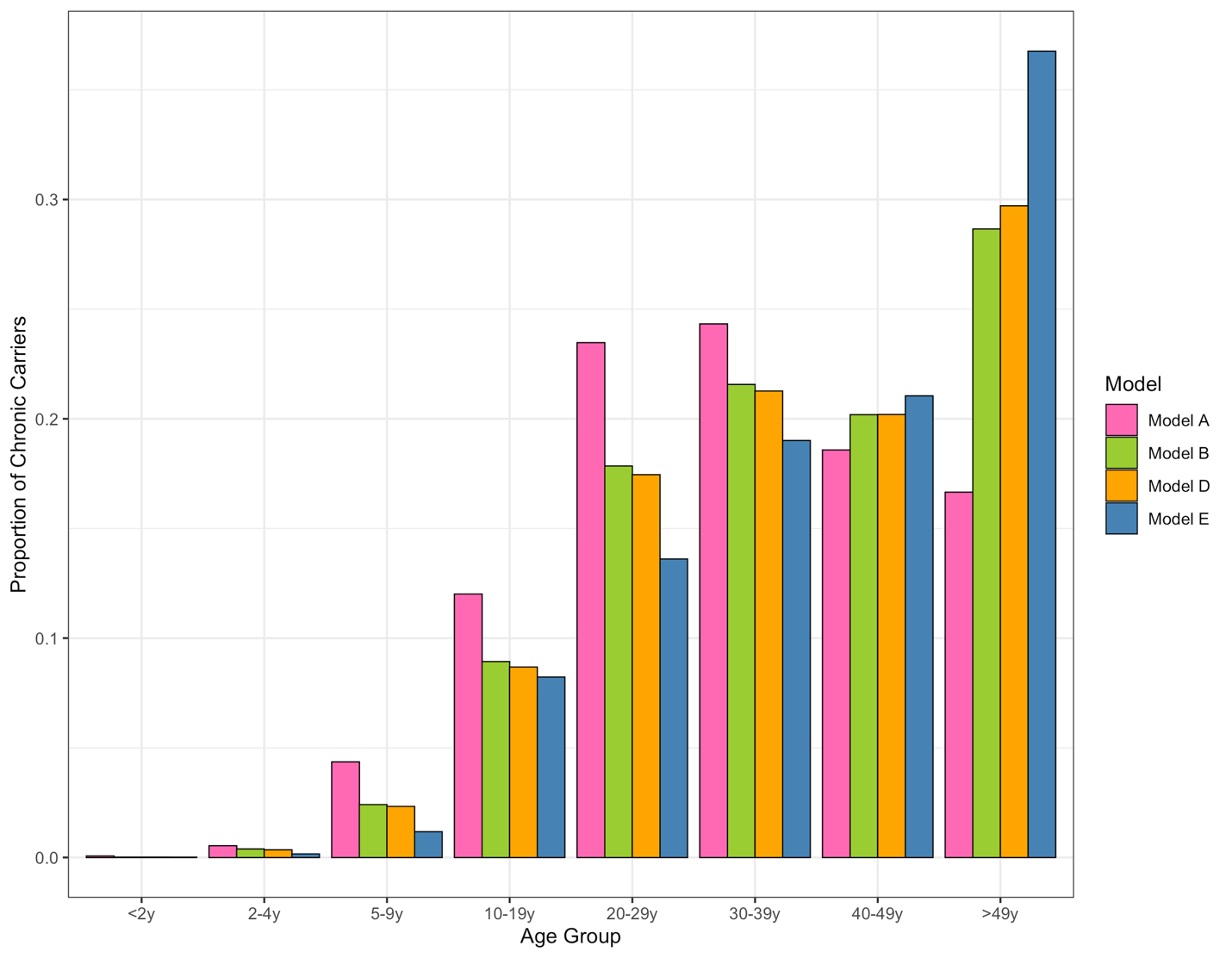


**Figure S6. Age distribution of chronic carriers before vaccine introduction for the four dynamic models.** Results are shown as the proportion of all chronic carriers that are in each age group, for each model (colored bars). The age distribution was similar at 10 years post-vaccine introduction.

**References**

[1] Sur D, von Seidlein L, Manna B, Dutta S, Deb AK, Sarkar BL, et al. The malaria and typhoid fever burden in the slums of Kolkata, India: data from a prospective community-based study. Trans R Soc Trop Med Hyg. 2006;100:725-33.<https://doi.org/10.1016/j.trstmh.2005.10.019>

[2] Mogasale V, Mogasale VV, Ramani E, Lee JS, Park JY, Lee KS, et al. Revisiting typhoid fever surveillance in low and middle income countries: lessons from systematic literature review of population-based longitudinal studies. BMC Infect Dis. 2016;16:35.<https://doi.org/10.1186/s12879-016-1351-3>

[3] Mogasale V, Maskery B, Ochiai RL, Lee JS, Mogasale VV, Ramani E, et al. Burden of typhoid fever in low-income and middle-income countries: a systematic, literature-based update with risk-factor adjustment. The Lancet Glob Health. 2014;2:e570-e80.<https://doi.org/10.1016/S2214-109X(14)70301-8>

[4] Mogasale V, Desai SN, Mogasale VV, Park JK, Ochiai RL, Wierzba TF. Case fatality rate and length of hospital stay among patients with typhoid Intestinal perforation in developing countries: a systematic literature review. PLOS ONE. 2014;9:e93784.<https://doi.org/10.1371/journal.pone.0093784>

[5] Sur D, Chatterjee S, Riewpaiboon A, Manna B, Kanungo S, Bhattacharya SK. Treatment cost for typhoid fever at two hospitals in Kolkata, India. J Health Popul Nutr. 2009;27:725-32.<https://doi.org/10.3329/jhpn.v27i6.4323>

[6] Salomon JA, Vos T, Hogan DR, Gagnon M, Naghavi M, Mokdad A, et al. Common values in assessing health outcomes from disease and injury: disability weights measurement study for the Global Burden of Disease Study 2010. The Lancet. 2012;380:2129-43.<https://doi.org/10.1016/S0140-6736(12)61680-8>

[7] Poulos C, Riewpaiboon A, Stewart JF, Clemens J, Guh S, Agtini M, et al. Cost of illness due to typhoid fever in five Asian countries. Trop Med Int Health. 2011;16:314-23.<https://doi.org/10.1111/j.1365-3156.2010.02711.x>

[8] Riewpaiboon A, Piatti M, Ley B, Deen J, Thriemer K, von Seidlein L, et al. Cost of illness due to typhoid Fever in Pemba, Zanzibar, East Africa. J Health Popul Nutr. 2014;32:377-85.<https://pubmed.ncbi.nlm.nih.gov/25395900>

[9] The World Bank. Life expectancy at birth, total (years) - India. 2005.<https://data.worldbank.org/indicator/SP.DYN.LE00.IN?end=2019&locations=IN&start=1960&view=chart>

[10] Portnoy A, Ozawa S, Grewal S, Norman BA, Rajgopal J, Gorham KM, et al. Costs of vaccine programs across 94 low- and middle-income countries. Vaccine. 2015;33 Suppl 1:A99-108.<https://doi.org/10.1016/j.vaccine.2014.12.037>

[11] EPIC Studies.<http://www.immunizationcosting.org>

[12] Ngabo F, Levin A, Wang SA, Gatera M, Rugambwa C, Kayonga C, et al. A cost comparison of introducing and delivering pneumococcal, rotavirus and human papillomavirus vaccines in Rwanda. Vaccine. 2015;33:7357-63.<https://doi.org/10.1016/j.vaccine.2015.10.022>

[13] Government of India Ministry of Health and Family Welfare. Universal Immunization Programme: Comprehensive Multi-Year Plan 2018-2022,.<https://www.who.int/immunization/programmes_systems/financing/countries/cmyp/india_cmyp_2013-17.pdf>

[14] Gandhi G, Lydon P. Updating the evidence base on the operational costs of supplementary immunization activities for current and future accelerated disease control, elimination and eradication efforts. BMC Public Health. 2014;14:67.<https://doi.org/10.1186/1471-2458-14-67>

[15] Shakya M, Colin-Jones R, Theiss-Nyland K, Voysey M, Pant D, Smith N, et al. Phase 3 efficacy analysis of a typhoid conjugate vaccine trial in Nepal. New Engl J Med. 2019;381:2209-18.<https://doi.org/10.1056/NEJMoa1905047>

[16] Shakya M, Voysey M, Theiss-Nyland K, Colin-Jones R, Pant D, Adhikari A, et al. Efficacy of typhoid conjugate vaccine in Nepal: final results of a phase 3, randomised, controlled trial. The Lancet Glob Health. 2021;9:e1561-e8.<https://doi.org/10.1016/S2214-109X(21)00346-6>
